# Accumulation of the delivered dose based on cone-beam CT and deformable image registration for non-small cell lung cancer treated with hypofractionated radiotherapy

**DOI:** 10.1186/s12885-020-07617-3

**Published:** 2020-11-16

**Authors:** Bin Wang, Da Quan Wang, Mao Sheng Lin, Shi Pei Lu, Jun Zhang, Li Chen, Qi Wen Li, Zhang Kai Cheng, Fang Jie Liu, Jin Yu Guo, Hui Liu, Bo Qiu

**Affiliations:** Department of Radiation Oncology, Sun Yat-sen University Cancer Center, State Key Laboratory of Oncology in South China, Collaborative Innovation Center for Cancer Medicine, Guangzhou, Guangdong China

**Keywords:** Non-small cell lung cancer, Hypofractionated radiotherapy, Accumulation, Deformable image registration

## Abstract

**Background:**

This study aimed to quantify the dosimetric differences between the planned and delivered dose to tumor and normal organs in locally advanced non-small cell lung cancer (LANSCLC) treated with hypofractionated radiotherapy (HRT), and to explore the necessity and identify optimal candidates for adaptive radiotherapy (ART).

**Methods:**

Twenty-seven patients with stage III NSCLC were enrolled. Planned radiation dose was 51Gy in 17 fractions with cone-beam CT (CBCT) acquired at each fraction. Virtual CT was generated by deformable image registration (DIR) of the planning CT to CBCT for dose calculation and accumulation. Dosimetric parameters were compared between original and accumulated plans using Wilcoxon signed rank test. Correlations between dosimetric differences and clinical variables were analyzed using Mann-Whitney U test or Chi-square test.

**Results:**

Patients had varied gross tumor volume (GTV) reduction by HRT (median reduction rate 11.1%, range − 2.9-44.0%). The V_51_ of planning target volume for GTV (PTV-GTV) was similar between original and accumulated plans (mean, 88.2% vs. 87.6%, *p* = 0.452). Only 11.1% of patients had above 5% relative decrease in V_51_ of PTV-GTV in accumulated plans. Compared to the original plan, limited increase (median relative increase < 5%) was observed in doses of total lung (mean dose, V_20_ and V_30_), esophagus (mean dose, maximum dose) and heart (mean dose, V_30_ and V_40_) in accumulated plans. Less than 30% of patients had above 5% relative increase of lung or heart doses. Patients with quick tumor regression or baseline obstructive pneumonitis showed more notable increase in doses to normal structures. Patients with baseline obstructive atelectasis showed notable decrease (10.3%) in dose coverage of PTV-GTV.

**Conclusions:**

LANSCLC patients treated with HRT had sufficient tumor dose coverage and acceptable normal tissue dose deviation. ART should be applied in patients with quick tumor regression and baseline obstructive pneumonitis/atelectasis to spare more normal structures.

**Supplementary Information:**

**Supplementary information** accompanies this paper at 10.1186/s12885-020-07617-3.

## Backgorund

Definitive radiotherapy combined with chemotherapy remains the main part of treatment for patients with unresectable locally advanced non-small cell lung cancer (LANSCLC) [1, 2]. Hypofractionated radiotherapy (HRT) has been demonstrated as an effective approach for dose escalation [3, 4]. The use of HRT is still challenging due to the close distance of target and organs at risk (OAR) in LANSCLC. The precise dose delivery has been achieved by the development of radiation technology, such as the simultaneous integrated boost intensity-modulated radiation therapy (SIB-IMRT) and on-board imaging guidance. However, it might be compromised by inter-fractional changes, which were caused by position shifts and anatomical changes of tumors and normal structures during treatment [5]. Such effects could be remarkable in HRT due to its higher fraction dose and significant tumor shrinkage [6].

Image-guided adaptive radiotherapy (ART) has been developed to account for the anatomic variations. It has been proposed that ART may be beneficial in reducing toxicities and allowing safe dose escalation in HRT. However, it is not widely used in clinic due to its time-consuming process and low cost-effectiveness. Therefore, it is essential to quantify the dosimetric differences between delivered and planned dose to tumor as well as OAR during HRT, in order to provide more information for proper patient selection. Cone beam CT (CBCT) has been routinely used for position verification and monitoring anatomical changes during radiotherapy for NSCLC patients [7]. However, due to poor resolution and inaccurate Housfield Unit (HU), the image quality of CBCT is typically insufficient for dose calculation and accumulation [8]. The virtual images can be generated by deforming the planning CT to the CBCT using deformable image registration (DIR), which provide real-time anatomy information with improved image quality [9]. This process enables the evaluation and accumulation of inter-fractional dose. Previous studies have confirmed that DIR is a validated tool for dose recalculation and accumulation among NSCLC patients treated with stereotactic body radiotherapy and conventional fractionated radiotherapy [10–13].

In this study, CBCT images and DIR technique were used to investigate the impact of inter-fractional anatomic changes on the dose delivered to tumor and normal structures in LANSCLC treated with HRT. By comparing the planned and delivered treatment dose, we aimed to explore the necessity and identify optimal candidates for ART.

## Methods

### Patients and treatment

Twenty-seven patients with stage III NSCLC were enrolled from a prospective clinical trial (NCT 02573506). Patients were immobilized and simulated according to the standard protocol for lung cancer [14]. A simulation four-dimensional CT (4DCT) scan was obtained with 3 mm thickness slices from the atlas to the second lumbar vertebra level to cover the whole neck and lung. The CT scan was performed with the Real-time Position Management system for respiratory gating (Varian Medical Systems, Palo Alto, CA). The CT images were automatically sorted into 10 phases corresponding to a single respiratory cycle. The maximum intensity projection (MIP) images were reconstructed from the 10 phases of images. The gross tumor volume (GTV) was defined as the visible primary tumor and positive lymph nodes on CT scans, which matched the volume of MIP images to account for respiratory motion. The clinical target volume (CTV) was delineated to include GTV plus a 0.5 cm margin and involved node regions. The planning target volumes for GTV (PTV-GTV) and CTV (PTV-CTV) were created by a uniform expansion of 0.5 cm surrounding the GTV and CTV, respectively. Thoracic RT was delivered using SIB-IMRT technique with a total dose of 51 Gy to PTV-GTV and 44.2 Gy to PTV-CTV in 17 daily fractions.

The study was reviewed and approved by Ethics Committee of our center. All patients provided signed informed consent for scientific usage of clinical data.

### CBCT acquisition

**Fig. 1:**
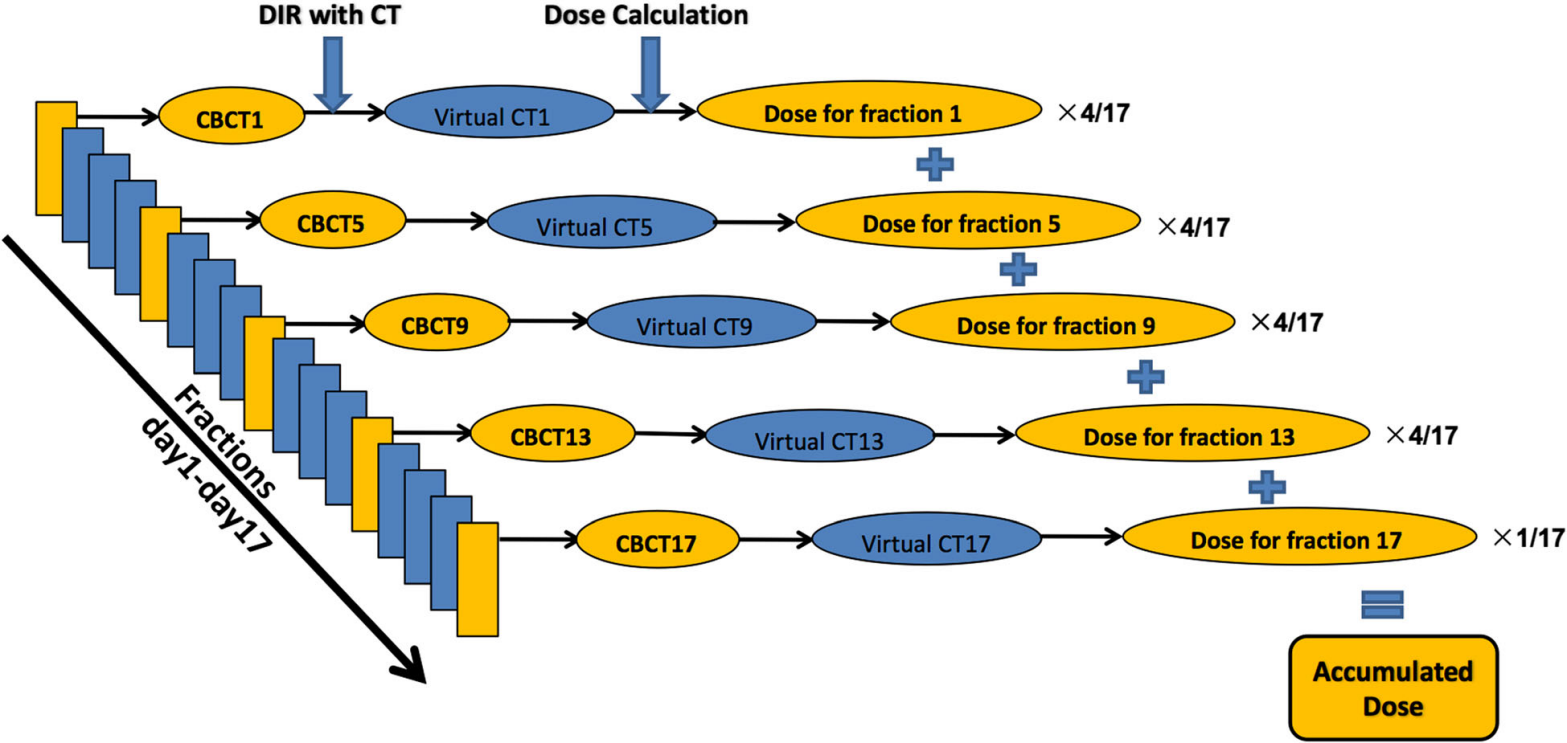
The study workflow (DIR: deformable image registration)

Patients underwent daily kilovoltage (kv)-based CBCT scan for set-up correction prior to the delivery of each radiation fraction. The CBCT scans were obtained using an Elekta VersaHD system (Elekta Lts, Crawley, UK). All scans were acquired under free breathing with patients in treatment position, which has accounted for internal movement because of the blurring of respiratory motion. The maximum scan length was 26.7 cm with a slice thickness of 3 mm. Other scanning parameters included 120 kV tube potential, 40 mA tuber current, 40 ms exposure time, 640 frames and 360° data collection. CBCT images obtained at fractions 1, 5, 9, 13, and 17 of treatment were selected for analyses in this study (Fig. 1).

### Virtual CT with deformable image registration

The planning CT was set as the reference image and the subsequent CBCTs as secondary images in Velocity software Version 3.2.0 (Velocity Medical Solutions, Atlanta, Georgia, USA). First, two sets of images were manually aligned using bonny structures as reference. Then, rigid registration was performed between planning CT and daily CBCT. After that, a correction procedure was performed to apply a fade correction and enhance low signal regions of the CBCT prior to the following registration. DIR was performed using modified B-spline deformable registration with mutual information-based matching [15]. The deformable vector field was manually reviewed to ensure the quality of DIR. Anatomical landmarks, e.g. bifurcation of airways and great vessels were used to evaluate the DIR accuracy. Once the deformation was reviewed to be acceptable by the operator, a virtual CT set was generated by deforming the planning CT according to the deformation vector field (DVF). The virtual CT was then exported to the treatment planning system (TPS) for dose calculations.

### Dose calculation based on virtual CT

The treatment beams from the original plan were copied and applied to the isocenters of virtual CT. Doses delivered by the original plan was recalculated on virtual CT images. It was noted that all beam parameters were consistent with the original treatment plan, including isocenter, control points and monitor units.

### Dose accumulation

Delivered dose was calculated for fraction 1, 5, 9, 13 and 17 using the virtual CTs respectively. The calculated dose on the virtual CT was then deformed back to the planning CT for dose accumulation using DVF obtained in the DIR between planning CT and daily CBCT. The cumulative dose was calculated as follows: D_sum_ = (D_frac1_ + D_frac5_ + D_frac9_ + D_frac13_) × 4 + D_frac17_. The following dosimetric parameters were recorded from the original and accumulated plan respectively: PTV-GTV: volume receiving ≥51 Gy (V_51_), dose covering at least 95% volume (D_95_), mean dose; Total lung: mean dose, volume receiving ≥20 Gy (V_20_) and 30 Gy (V_30_); Esophagus: maximum dose (D_max_) and mean dose; Heart: mean dose, volume receiving ≥30 Gy (V_30_) and 40 Gy (V_40_).

### Statistical analysis

Dosimetric parameters were presented as mean ± SD. Relative dosimetric difference between original and accumulated plan was calculated as: (accumulated plan-original plan)/original plan, and was presented as median value with range. In this study, a relative change ≥5% in dosimetric parameters was considered clinically meaningful. Tumor volume and dosimetric parameters were compared between original and accumulated plans using Wilcoxon signed rank test. Correlations between dosimetric differences and clinical variables, including sex, age, tumor location, presence of bulky mediastinal lymph node, cTNM stage, presence of obstructive pneumonitis/atelectasis and tumor regression, were analyzed using Mann-Whitney U test or Chi-square test. All tests were two-sided, and p<0.05 was considered statistically significant. All the analysis was performed with SPSS (Version 22.0).

## Results

### Patient characteristics

The clinical characteristics of 27 patients were summarized in Table 1. Of all patients, the median age at diagnosis was 61 years (range, 42–72). Five cases (18.5%) were females and 22 (81.5%) were males. Twelve patients (44.4%) had stage IIIA and 15 (55.6%) had stage IIIB diseases. There were 22 cases (81.5%) with central type tumor and 5 cases (18.5%) with peripheral type. Baseline obstructive pneumonitis/atelectasis was observed in 11 patients (40.7%). Mediastinal shift occurred in 3 patients (11.1%) during the course of treatment.

**Table 1:** Patients’ clinical characteristics (*n* = 27)

characteristics	*n* (%)
Age (median, range)	61 (42–72)
Sex	
Female	5 (18.5%)
Male	22 (81.5%)
Primary tumor location	
Left	13 (48.1%)
Right	12 (44.4%)
Mediastinum	2 (7.4%)
Type of lung tumor	
Central	22 (81.5%)
Peripheral	5 (18.5%)
cTNM stage	
IIIA	12 (44.4%)
IIIB	15 (55.6%)
Bulky mediastinal lymph node	
Yes	6 (22.2%)
No	21 (77.8%)
Obstructive pneumonitis/atelectasis	
Yes	11 (40.7%)
No	16 (59.3%)
Tumor being adjacent to esophagus	
Yes	12 (44.4%)
No	15 (55.6%)
Mediastinal shift	
Yes	3 (11.1%)
No	24 (88.9%)

### Gross tumor volume changes

**Fig. 2:**
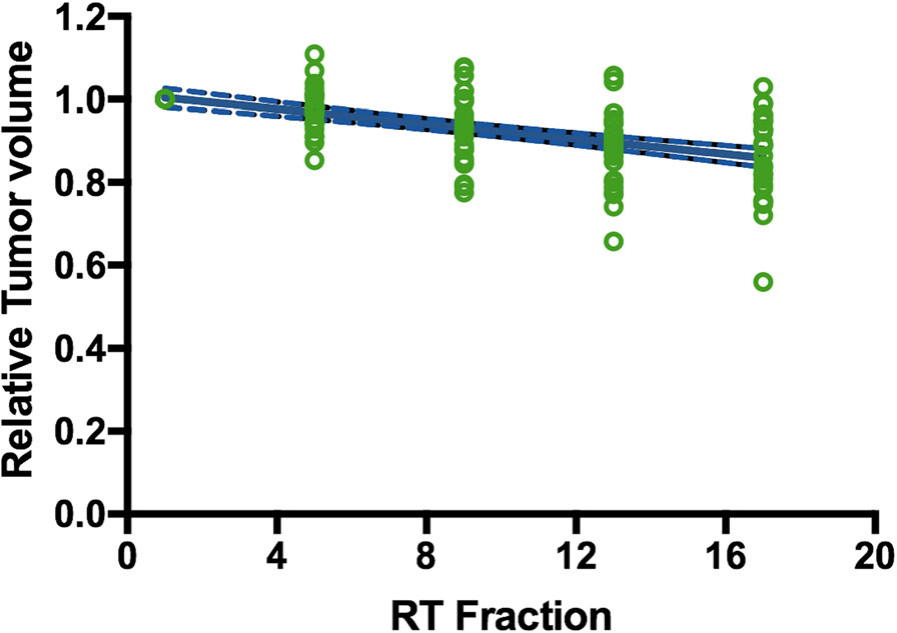
The reduction in gross tumor volume during the course of radiotherapy (RT: radiotherapy)

As shown in Fig. 2, there was significant tumor shrinkage through the course of treatment. Average GTV volume reduced from 145.1cm^3^ at fraction 1 to 117.9 cm^3^ at fraction 17, and the difference was statistically significant (p<0.001). The median relative GTV reduction was 2.1, 6.2, 10.6, and 11.1% from start to fraction 5, 9, 13 and 17 of therapy, respectively. Greater tumor shrinkage was achieved from fraction 5 to 13, compared with that from fraction 1 to 5 or fraction 13 to 17 (8.5% vs. 2.1% vs. 0.5%). Detailed information about GTV volume change was shown in Table 2.

**Table 2:** Gross tumor volumes at different fractions of radiotherapy

Variables	Fraction 1	Fraction 5	Fraction 9	Fraction 13	Fraction 17
Mean GTV (cm^3^)	145.1 ± 159.8	139.5 ± 148.9	131.2 ± 140.2	122.9 ± 127.4	117.9 ± 122.2
Median GTV (cm^3^)	76.5 (10.4–587.2)	73.7 (10.3–501.34)	71.5 (11.2–503)	69.5 (9.5–475.8)	66.1 (10.3–468.1)
Median relative GTV volume	1.0	0.979 (0.854–1.108)	0.938 (0.775–1.077)	0.894 (0.658–1.057)	0.889 (0.560–1.029)
Relative GTV Reduction	0	2.1%(−10.8–14.6%)	6.2%(−7.7–22.5%)	10.6%(−5.7–34.2%)	11.1%(−2.9–44%)

### Dosimetric differences between original and accumulated plans

As demonstrated in Table 3, PTV-GTV coverage were similar between original and accumulated plans. Average V_51_ of PTV-GTV were 88.2 and 87.6% in original and accumulated plans (*p* = 0.452), with average D_95_ of 48.1 Gy and 48.0 Gy (*p* = 0.781), respectively. In accumulated plans, only 3 patients (11.1%) showed a relative reduction in V_51_ above 5% when compared with original plans.

**Table 3:** The comparison of dosimetric parameters between original and accumulated plans

	Original plan (mean ± SD)	Accumulated plan (mean ± SD)	*P* value	Relative change (median, range)	Above 5% relative reduction/increase*
PTV-GTV					
V_51_ (%)	88.2 ± 6	87.6 ± 6.3	0.452	−0.1% (−10–6%)	3 (11.1%)
D_95_ (Gy)	48.1 ± 2.0	48.0 ± 2.3	0.781	0.1% (−7.6–5%)	1 (3.7%)
D_mean_ (Gy)	52.6 ± 0.5	52.6 ± 0.6	0.873	0.04% (−1–1%)	0
Total lung					
V_20_ (%)	29.5 ± 4.2	30.1 ± 4.1	**0.001**	1.6% (−3–9%)	3 (11.1%)
V_30_ (%)	19.9 ± 4.4	20.4 ± 4.4	**0.004**	1.9% (−3–16%)	6 (22.2%)
D_mean_ (Gy)	16.8 ± 2.1	17.0 ± 2.1	**0.001**	1.4% (−2–5%)	1 (3.7%)
Esophagus					
D_max_ (Gy)	47.4 ± 1.3	49.5 ± 1.8	**<0.001**	4.1% (−4–15%)	10 (37.0%)
D_mean_ (Gy)	26.6 ± 4.2	27.1 ± 4.0	**0.002**	1.4% (−2–5%)	1 (3.7%)
Heart					
V_30_ (%)	13.0 ± 10.1	12.9 ± 10.9	0.675	−17.4% (−48.6–16.4%)	7 (25.9%)
V_40_ (%)	5.2 ± 5.0	4.9 ± 5.1	0.168	−7.1% (−73.9–26.4%)	8 (29.6%)
D_mean_ (Gy)	13.0 ± 6.7	13.1 ± 7.0	0.558	1% (−1.9–12.4%)	5 (18.5%)

#Relative change = (Accumulated plan-Original plan)/Original plan×100%

*Number of patients who had above 5% reduction in PTV-GTV dosage or above 5% increase in OARs dosage

Besides, the D_95_ and V_51_ of PTV-GTV were compared between fraction plans (based on CBCT images at fraction 1, 5, 9, 13 or 17) and original plan, which was shown in Supplementary Table 1. The results indicated that the percentage of patients obtaining a reduction above 5% in D_95_ was higher at fraction 5 compared with that at fraction 9, 13 or 17 (18.5% vs. 11.1% vs. 11.1% vs. 11.1%, *p* = 0.448); the percentage of patients obtaining a reduction above 5% in V_51_ was higher at fraction 5 compared with that at fraction 9, 13 or 17 (22.2% vs. 14.8%, p = 0.448; 22.2% vs. 11.1%, *p* = 0.278; 22.2% vs. 7.4%, *p* = 0.129); the percentage of patients obtaining a reduction above 5% in V_51_ was higher at fraction 9 compared with that at fraction 13 or 17 (14.8% vs. 11.1%, *p* = 0.688; 14.8% vs. 7.4%, *p* = 0.391).

Slight, although statistically significant differences were observed in the lungs’ dosage between original and accumulated plans. The total lung V_20_ increased from 29.5% ± 4.2% in original plans to 30.1% ± 4.1% in accumulated plans (*p* = 0.001), with a median relative increase of 1.6% (range, − 3-9%). The total lung V_30_ increased from 19.9% ± 4.4% in original plans to 20.4% ± 4.4% in accumulated plans (*p* = 0.004), with a median relative increase of 1.9% (range, − 3-16%). Significant difference in the mean lung dose was also observed in accumulated plans (17.0 ± 2.1 vs. 16.8 ± 2.1, p = 0.001), with a median relative increase of 1.4% (range, − 2-5%). In accumulated plans, there are 3 (11.1%) and 6 (22.2%) patients obtained a relative increase above 5% in the lung V_20_ or V_30_ when compared with original plans.

For the esophagus, an increase was observed in mean (27.1 ± 4.0 vs. 26.6 ± 4.2, *p* = 0.002) and maximal doses (49.5 ± 1.8 vs. 47.4 ± 1.3, p<0.001) in accumulated plans, with median relative increase of 1.4% (range, − 2-5%) and 4.1% (range, − 4-15%), respectively. Ten patients (37.0%) obtained a relative increase above 5% in maximal dose.

There was no significant difference in the heart mean dose (13.0 ± 6.7 vs. 13.1 ± 7.0, *p* = 0.558), V_30_ (13.0 ± 10.1 vs.12.9 ± 10.9, *p* = 0.675) or V_40_ (5.2 ± 5.0 vs. 4.9 ± 5.1, *p* = 0.168) between original and accumulated plans. Seven patients (25.9%) achieved above 5% relative increase in the heart V_30_ and 8 (29.6%) achieved that in V_40_.

### Predictors of dosimetric differences

**Fig. 3:**
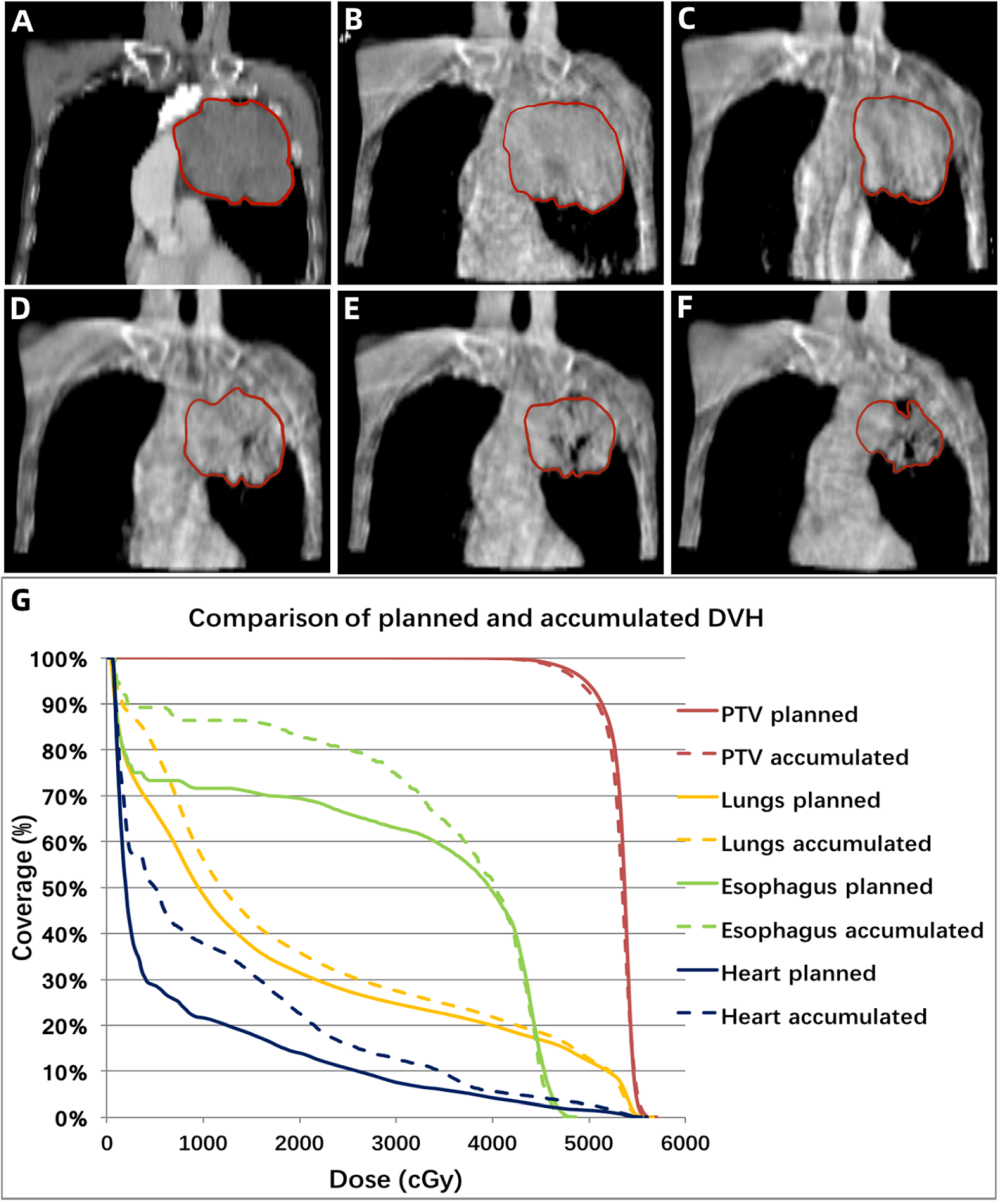
Example case with quick tumor regression during radiotherapy **a**-**f** and the comparison of dose volume histogram (DVH) between original and accumulated plan (G; Solid line: original plan, Dashed line: accumulated plan). The GTV volume was estimated in contrast-enhanced simulation CT **a**, and CBCT at fraction 1 **b**, fraction 5 **c**, fraction 9 **d**, fraction 13 **e** and fraction 17 **f** of radiotherapy

Correlations between dosimetric differences and clinical variables were analyzed. Based on the magnitude of tumor shrinkage, 27 patients were divided into two groups: quick tumor regression and slow tumor regression groups. Quick tumor regression was defined as a tumor shrinkage by over 11.1% (the median value of relative GTV reduction), otherwise it was defined as slow. Patients with quick tumor regression showed more notable relative increase in the total lung V_20_ (median, 2.7% vs. 0.5%, *p* = 0.006), V_30_ (median, 3.5% vs. 0.2%, *p* = 0.022) and mean dose (median, 2.2% vs. 0.2%, *p* = 0.009) in accumulated plans than those who did not. Besides, in quick tumor regression group, more patients were observed to have a relative increase above 5% in the lung V_20_ (23.1% vs. 0, *p* = 0.061) and V_30_ (38.5% vs. 7.1%, *p* = 0.055). Tumor regression also correlated with the differences of the esophagus mean dose (2.2% vs. 0.2%, p = 0.009), but not maximal dose (4.0% vs 5.0%, *p* = 0.239). No correlation was found between tumor regression and heart doses change (Table 4). Figure 3 shows a typical case with quick tumor regression. The doses to OARs increased markedly in accumulation plan compared with original plan.

**Table 4:** Dosimetric changes affected by tumor regression and baseline obstructive pneumonitis/atelectasis

Variables	Relative change (median) #		Proportion of patients achieving above 5% reduction/increase*	
	Tumor regression [quick(*n* = 13) vs. slow(*n* = 14)]	Obstructive pneumonitis/atelectasis [Yes(*n* = 11) vs. No(*n* = 16)]	Tumor regression [quick(*n* = 13) vs. slow(*n* = 14)]	Obstructive pneumonitis/atelectasis [Yes(*n* = 11) vs. No(*n* = 16)]
PTV-GTV				
V_51_	0.2% vs. -0.7%(*P* = 0.185)	−0.3% vs. -0.1%(*P* = 0.394)	7.7% vs. 14.3%(*P* = 0.593)	10% vs. 11.8%(*P* = 0.89)
D_95_	0.3% vs. -0.1%(*P* = 0.867)	−0.05% vs. 0.4%(*P* = 0.942)	0% vs. 7.1%(*P* = 0.335)	10% vs. 0%(*P* = 0.192)
Total lung				
D_mean_	**2.2% vs. 0.2%(*P*** **= 0.009)**	**2.3% vs. 0.4%(*P*** **= 0.023)**	7.7% vs. 0%(*P* = 0.299)	9.1% vs. 0%(*P* = 0.228)
V_20_	**2.7% vs. 0.5%(*P*** **= 0.006)**	3.3% vs. 0.9%(*P* = 0.110)	23.1% vs. 0(*P* = 0.061)	9.1% vs. 12.5%(*P* = 0.786)
V_30_	**3.5% vs. 0.2%(*P*** **= 0.022)**	**3.9% vs. 0.2%(*P*** **= 0.039)**	38.5% vs. 7.1%(*P* = 0.055)	36.4% vs. 12.5%(*P* = 0.150)
Esophagus				
D_mean_	**2.2% vs. 0.2%(*P*** **= 0.009)**	**2.3% vs. 0.4%(*P*** **= 0.023)**	7.7% vs. 0%(*P* = 0.299)	9.1% vs. 0%(*P* = 0.228)
D_max_	4.0% vs 5.0%(*P* = 0.239)	3.2% vs. 4.8%(*P* = 0.272)	23.1% vs. 50%(*P* = 0.155)	27.3% vs. 43.8%(*P* = 0.393)
Heart				
D_mean_	0.1% vs. 0.8%(*P* = 0.488)	3.9% vs. 0.2%(*P* = 0.251)	**38.5% vs. 0%(*P*** **= 0.012)**	**36.4% vs. 6.3%(*P*** **= 0.046)**
V_30_	−5.2% vs. -1.7%(*P* = 0.375)	8.1% vs. -5.2%(*P* = 0.162)	38.5% vs. 14.3%(*P* = 0.16)	**54.5% vs. 6.3%(*P*** **= 0.006)**
V_40_	−16.1% vs. -5.7%(*P* = 0.458)	2.1% vs. -16.1%(*P* = 0.272)	30.8% vs. 28.6%(*P* = 0.902)	45.5% vs. 18.8%(*P* = 0.143)

#Relative change = (Accumulated plan-Original plan)/Original plan×100%

*proportion of patients who had above 5% reduction in PTV-GTV dosage or above 5% increase in OARs dosage

In patients with baseline obstructive pneumonitis/atelectasis, significantly greater relative increase in the total lung mean dose (median, 2.3% vs. 0.4%, *p* = 0.023), lung V_30_ (median, 3.9% vs. 0.2%, *p* = 0.039) and esophagus mean dose (median, 2.3% vs. 0.4%, p = 0.023) was observed compared to those without. Greater increase was also observed in the total lung V_20_ (median, 3.3% vs. 0.9%, *p* = 0.110), heart mean dose (median, 3.9% vs. 0.2%, *p* = 0.251), heart V_30_ (median, 8.1% vs. -5.2%, *p* = 0.162) and heart V_40_ (median, 2.1% vs. -16.1%, *p* = 0.272), despite none of them was statistically significant. Furthermore, in the group with baseline obstructive pneumonitis/atelectasis, more patients were observed to gain above 5% relative increase in heart V_30_ (54.5% vs. 6.3%, *p* = 0.006) and heart mean dose (36.4% vs. 6.3%, *p* = 0.046) (Table 4).

**Fig. 4:**
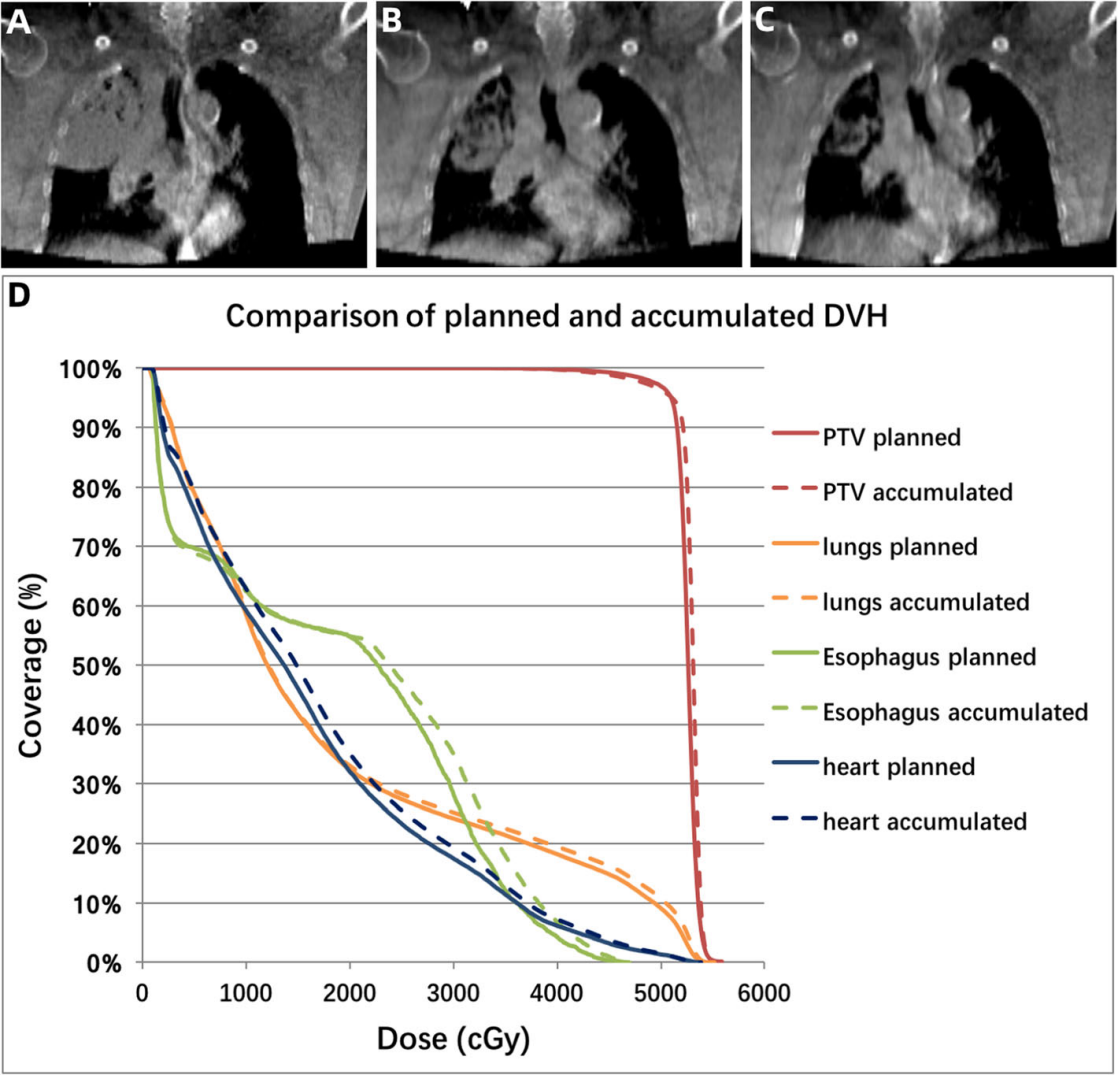
A typical case with mediastinum shifting to the ipsilateral side as tumor regressed. **a** CBCT scan performed at fraction 1 of radiotherapy; **b**-**c** CBCT scans at fraction 9 and 17 revealed a mediastinal shift to the ipsilateral side; **d** the comparison of dose volume histogram (DVH) between original and accumulated plan (Solid line: original plan, Dashed line: accumulated plan)

**Fig. 5:**
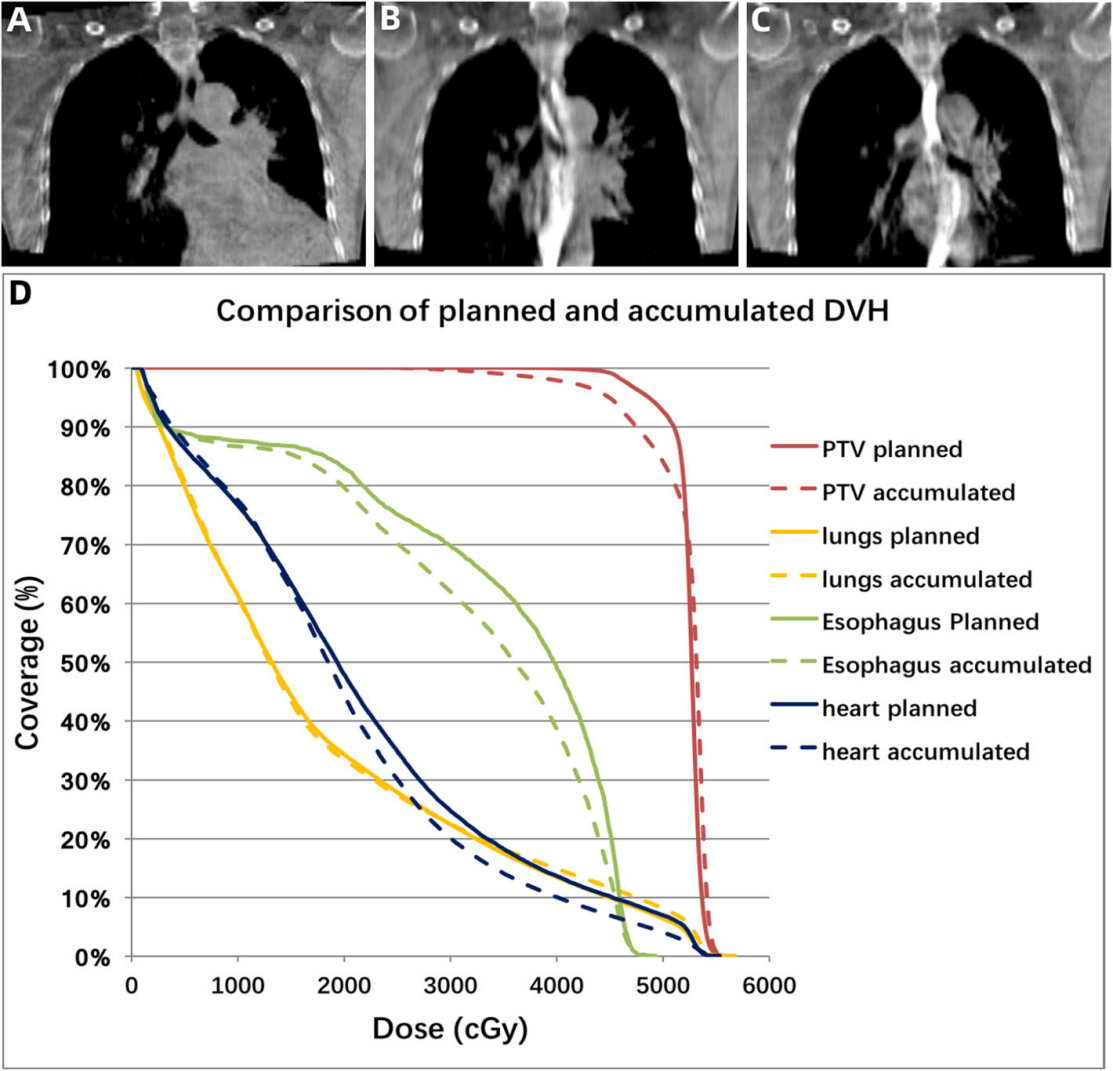
A typical case with mediastinum shifting to contralateral side as lung re-expanded. **a** CBCT scan performed at fraction 1 of radiotherapy; **b**-**c**. CBCT scans at fraction 9 and 17 revealed a mediastinal shift to the contralateral side; **d** the comparison of dose volume histogram (DVH) between original and accumulated plan (Solid line: original plan, Dashed line: accumulated plan)

Mediastinal shift to the ipsilateral side during treatment was observed in two cases with baseline obstructive pneumonitis. Figure 4 showed a typical case with mediastinum shifting to the ipsilateral side as tumor regressed. Increased doses to normal structures were observed, including total lung, esophagus and heart. On the contrary, mediastinal shift to the contralateral side during treatment was observed in a case with baseline obstructive atelectasis (Fig. 5). A notable decrease of 10.3% were observed in V_51_ of PTV-GTV. As for normal structures, decrease was also obtained in doses to total lung, esophagus and heart.

None of other clinical variables, including sex, age, tumor location, presence of bulky mediastinal lymph node or TNM stage, was found to be predictive of the dose differences of PTV-GTV or OARs between original and accumulative plans.

## Discussion

Previous studies showed that HRT with a dedicated technique allows safety dose escalation, minimizing the effect of tumor repopulation and improving local control [16–18]. Compared to SBRT, a more fractionated approach was applied in modestly HRT for LANSCLC, and it mostly ranged from 12 to 20 fractions [19–21]. In this study, we accumulated the delivered dose using CBCT and DIR in patients without ART, and compared the delivered dose to planned dose. Our results indicated that, for most patients, the differences between delivered and planned dose were clinically acceptable. The median relative reduction in V_51_ of PTV-GTV was only 0.1%. In terms of normal tissues, although statistically significant difference was observed in lung and esophagus doses between original and accumulated plans, the median relative increase in most metrics was very limited (less than 2%), except for maximal esophagus dose with a median relative increase of 4.1%. Considering that a 5% relative difference in dosimetric parameters would be clinically meaningful, we analyzed the number of patients who obtained above 5% relative reduction in PTV-GTV coverage or 5% relative increase in normal organs’ dosage. Overall, only 11.1% of patients were observed to have above 5% relative decrease in V_51_ of PTV-GTV. Meanwhile, less than 30% of patients achieved above 5% relative increase in lung or heart doses. According the results, non-ART strategy was acceptable for most patients treated by HRT, and only a small proportion of patients might be in need of ART during the treatment.

For these reasons, the changes of GTV during treatment course need to be investigated. Previous studies found that the mean GTV reduction was about 19–51.1% in patients treated by conventional fractionated radiotherapy [22–25]. A less marked reduction in tumor volume was obtained during SBRT due to the short irradiation course, with the mean GTV reduction of 12.8–32% [26, 27]. In current study, we detected a gradual shrinking of tumor volume over the course of treatment with median reduction of 11.1%, which suggested only a fraction of patients could benefit from ART. The extent of tumor regression has been used to identify candidates for ART in previous studies [28, 29]. Woodford et al. found that ART was required when a patient had a greater than 30% reduction in tumor volume within the first 20 fractions [28]. Therefore, we identified the correlation between tumor regression and the dosimetric difference. Patients with GTV reduction over 11.1% had more significant increase in lung and esophagus doses in the accumulated plans. It supported the idea that the magnitude of GTV reduction might be used as an indicator for plan adaption. Previous study explored the optimal time point for plan adaption among NSCLC treated by modestly HRT (10–12 fractions). And the treatment midpoint was considered as a favorable time for plan adaptation, since notable tumor response and sufficient remaining fractions were both obtained at present [29]. Similarly, in our study, notable tumor response was achieved from fraction 5 to 13. In order to further identify the optimal time point in the course of radiotherapy for re-planning, we analyzed the number of patients who obtained a reduction above 5% in D_95_ and V_51_ of PTV-GTV when comparing the fraction plan at different time point of treatment course with the original plan. The results showed that the percentage of patients obtaining a reduction above 5% in D_95_ and V_51_ of PTV-GTV were higher at fraction 5 compared with that at fraction 9, 13, or 17. Besides, a higher percentage of patients obtaining >5% reduction in V_51_ of PTV-GTV was also observed at fraction 9 compared with that at fraction 13 or 17. Taking together the changes in GTV volume, D_95_ and V_51_ of PTV-GTV at different time points, we recommended the fraction 9 in the course of radiotherapy as the time point for re-planning.

For lung cancer patients with obstructive pneumonitis or atelectasis before radiotherapy, the volume and position of lung tissue could progressively change with treatment [30]. Previous studies reported that the resolution rate of atelectasis was 38–90% with thoracic radiotherapy in lung cancer patients [31–33]. Great deviation in dose distribution might occur when a patient experiences great changes in the lung tissue. Moller et al. found that ART was required in 70% of lung cancer patients with atelectasis [34]. Our study indicated that patients with baseline obstructive pneumonitis/atelectasis achieved more notable increase in total lung, esophagus and heart dose than those without. In addition, 54.5 and 45.5% of patients with obstructive pneumonitis/atelectasis were observed to gain above 5% relative increase in heart V30 and V40, respectively. The results suggested that patients with baseline obstructive pneumonitis or atelectasis might benefit more from ART. And frequent CBCT scans should be performed in order to enable the immediate access to ART when great change in lung volume and position was obtained.

Other than the change of lung volume and position, mediastinal shift remained crucial for dose deviation of OARs for patients with baseline obstructive pneumonitis/atelectasis. Mediastinal shift could occur as the regression of tumor and resolution of pneumonitis/atelectasis during radiotherapy. In fact, it is difficult to directly quantify the movement of mediastinum, since it consisted of multiple organs and structures. Esophagus, as a longitudinal organ located in the middle of mediastinum, could largely reflect the motion of mediastinum. In previous study, we quantified the interfractional motion of esophagus during fractionated radiotherapy for 36 patients with locally advanced non-small cell lung cancer [35]. Oral barium sulfate was administrated during CBCT to help localize the esophagus. The results indicated that the motion in right-left direction were − 24.0 ~ 11.9 mm, − 27.8 ~ 13.1 mm and − 36.3 ~ 10.4 mm for proximal, middle and distal thoracic esophagus respectively. These data could to some extent reflect the movement of mediastinum. In the present study, the presence of upper and middle mediastinum shift was defined as a right-left motion of esophagus above 10 mm, which had occurred in three patients. The occurrence of mediastinal shift might result in inadequate tumor dose coverage or increased normal tissue doses [36]. In patients with baseline obstructive pneumonitis, the mediastinum usually deviated to the ipsilateral side with the remission of tumor, which resulted in the increased exposure of mediastinal structures. However, for patients with baseline obstructive atelectasis, the mediastinum might deviate to the contralateral side because of the re-expansion of lung after treatment. The inadequate dose coverage of target volume remained the main concern for these patients. Despite the example size was limited, we still supposed that the plan adaption should be taken into account for patients with baseline obstructive atelectasis and mediastinal shift.

The limitation of the study is that the dose accumulation was not based on single delivered dose of each fraction, which may have an impact on the accuracy of dose accumulation. We chose fractionated dose at five time-points (fraction 1, 5, 9, 13 and 17) for dose calculation and accumulation, which can largely reflect the actual dose that patient has received. Tissue appearance and disappearance (TAD) (such as the tumor regression) might also hamper the accuracy of DIR between CBCT and reference CT [37]. While it was still an open problem to be addressed by the radiotherapy community, measures had been taken to minimize the impact of TAD and lung tissue changes on the DIR accuracy. With careful inspection of the DIR results using anatomical landmarks, dose accumulation uncertainties could be reduced to the clinical acceptable level [38].

## Conclusions

In summary, most patients with LANSCLC had sufficient tumor dose coverage and acceptable normal tissue dose deviation without ART in the treatment of modestly HRT. Quick tumor regression, baseline obstructive pneumonitis/atelectasis during treatment are potential indicators for the need of ART. Daily CBCT scans prior to treatment was essential for patients treated by HRT, which enabled the timely detection of anatomical change for tumors and normal structures. To assess the advantage of ART based on the above factors in prospective studies are warranted.

## Supplementary Information

**Additional file 1: Supplementary table 1.** The comparison of PTV-GTV D_95_ and V_51_ between the fraction plans at different time point of treatment course with the original plan.

## Data Availability

The datasets used and/or analyzed during the current study are available from the corresponding author on reasonable request.

## Abbreviations

LANSCLC: Locally advanced non-small cell lung cancer
HRT: Hypofractionated radiotherapy;
OAR: Organs at risk
SIB-IMRT: Simultaneous integrated boost intensity-modulated radiation therapy
ART: Adaptive radiotherapy
CBCT: Cone beam CT
DIR: Deformable image registration
4DCT: Four-dimensional CT
MIP: Maximum intensity projection
DVF: Deformation vector field
TPS: Treatment planning system
GTV: Gross tumor volume
CTV: Clinical target volume
PTV: Planning target volume
DVH: Dose volume histogram
