# Supplementary material for: Accumulation of the delivered dose based on cone-beam CT and deformable image registration for non-small cell lung cancer treated with hypofractionated radiotherapy
Source: BMC Cancer. 2020 Nov 16;20:1112. doi: 10.1186/s12885-020-07617-3 (PMC7670776; doi:10.1186/s12885-020-07617-3)
Supplement: Supplementary file 1 — Additional file 1: Supplementary table 1. The comparison of PTV-GTV D95 and V51 between the fraction plans at different time point of treatment course with the original plan. [file 12885_2020_7617_MOESM1_ESM.docx]

Supplementary table 1. The comparison of PTV-GTV D_95_ and V_51_ between the fraction plans at different time point of treatment course with the original plan

| Fractions | Number of patients achieving above 5% reduction [Original plan- Fra plan)/Original plan✖100%] | | |  |
| --- | --- | --- | --- | --- |
|  | PTV-GTV D_95_ | P value | PTV-GTV V_51_ | P value |
| Fraction 1 | 3 (11.1%) | 0.715 | 4 (14.8%) | 0.222 |
| Fraction 5 | 5 (18.5%) |  | 6 (22.2%) |  |
| Fraction 9 | 3 (11.1%) | 0.448 (Fra 5 vs. 9) | 4 (14.8%) | 0.488 (Fra 5 vs. 9) |
| Fraction 13 | 3 (11.1%) | 0.448 (Fra 5 vs. 13) | 3 (11.1%) | 0.278 (Fra 5 vs. 13) |
| Fraction 17 | 3 (11.1%) | 0.448 (Fra 5 vs. 17) | 2 (7.4%) | 0.129 (Fra 5 vs. 17) |
